# Requirements for *Pseudomonas aeruginosa* Acute Burn and Chronic Surgical Wound Infection

**DOI:** 10.1371/journal.pgen.1004518

**Published:** 2014-07-24

**Authors:** Keith H. Turner, Jake Everett, Urvish Trivedi, Kendra P. Rumbaugh, Marvin Whiteley

**Affiliations:** 1 Department of Molecular Biosciences, Institute of Cellular and Molecular Biology, Center for Infectious Disease, The University of Texas at Austin, Austin, Texas, United States of America; 2 Department of Surgery, Texas Tech University Health Sciences Center, Lubbock, Texas, United States of America; The University of Texas Health Science Center at Houston, United States of America

## Abstract

Opportunistic infections caused by *Pseudomonas aeruginosa* can be acute or chronic. While acute infections often spread rapidly and can cause tissue damage and sepsis with high mortality rates, chronic infections can persist for weeks, months, or years in the face of intensive clinical intervention. Remarkably, this diverse infectious capability is not accompanied by extensive variation in genomic content, suggesting that the genetic capacity to be an acute or a chronic pathogen is present in most *P. aeruginosa* strains. To investigate the genetic requirements for acute and chronic pathogenesis in *P. aeruginosa* infections, we combined high-throughput sequencing-mediated transcriptome profiling (RNA-seq) and genome-wide insertion mutant fitness profiling (Tn-seq) to characterize gene expression and fitness determinants in murine models of burn and non-diabetic chronic wound infection. Generally we discovered that expression of a gene *in vivo* is not correlated with its importance for fitness, with the exception of metabolic genes. By combining metabolic models generated from *in vivo* gene expression data with mutant fitness profiles, we determined the nutritional requirements for colonization and persistence in these infections. Specifically, we found that long-chain fatty acids represent a major carbon source in both chronic and acute wounds, and *P. aeruginosa* must biosynthesize purines, several amino acids, and most cofactors during infection. In addition, we determined that *P. aeruginosa* requires chemotactic flagellar motility for fitness and virulence in acute burn wound infections, but not in non-diabetic chronic wound infections. Our results provide novel insight into the genetic requirements for acute and chronic *P. aeruginosa* wound infections and demonstrate the power of using both gene expression and fitness profiling for probing bacterial virulence.

## Author Summary

Soft tissue infections, such as those in burns, bed sores, and diabetic ulcers, are a significant healthcare and economic burden in the developed and developing world. The opportunistic pathogen *P. aeruginosa* can cause both acute and chronic infections, and the trajectory of these two types of infections is vastly different. We used high-throughput sequencing to profile *P. aeruginosa* genome-wide gene expression and mutant fitness during mouse model acute and non-diabetic chronic wound infections. Using these data, we show that wounds are nutrient-rich growth environments in which long-chain fatty acids are a primary source of carbon and energy. We also show that the ability to travel along spatio-chemical gradients by chemotaxis is critical for bacterial fitness and virulence in acute but not chronic infections. Our results demonstrate the utility of simultaneous mutant fitness and gene expression profiling to discover critical functions in complex growth environments.

## Introduction

Infections caused by opportunistic bacterial pathogens are a primary cause of morbidity and mortality in both the developed and developing world. These infections are often characterized by robust growth of the pathogen in the infection site and increasingly high resistance to antibiotic treatment. The opportunistic pathogen *Pseudomonas aeruginosa* is responsible for a wide range of infections in immunocompromised hosts [1]. Among the most significant of these infections are those localized to soft tissues, including chronic and burn wounds [1], [2]. Chronic wounds are defined as wounds that have “failed to proceed through an orderly and timely process to produce anatomic and functional integrity, or proceeded through the repair process without establishing a sustained anatomic and functional result” [3]. Chronic wounds include pressure ulcers (bed sores), diabetic ulcers, venous ulcers, and arterial ulcers, and affect approximately 5–7 million people per year in the US at a cost of $10–20 billion per year [4]. Infections in burn wounds also carry a heavy medical and economic burden not only in the developed world, but also in the developing world, where 70% of burns affect children, and mortality in patients with burns covering >40% total body surface area approaches 100% [5], [6]. Interestingly, burn infections caused by *P. aeruginosa* often deteriorate rapidly and lead to systemic spread and death within days or weeks, yet *P. aeruginosa* chronic wound infections persist for much longer with little associated mortality [5]. As this difference in infection trajectory is not thought to be a result of colonization with specific *P. aeruginosa* strains, the mechanisms underlying this difference remain a mystery. One possibility is that the type of injury impacts key features of the host environment, such as the immune response, and that this dictates disease progression [7]. A second, non-mutually exclusive possibility is that *P. aeruginosa* physiology and gene expression is different in chronic and acute wounds. In this work, we set out to address this second possibility using genomic methods.

Phenotypes thought to be associated with acute or chronic *P. aeruginosa* infections have been extensively studied *in vitro*, and much is known about the genes responsible for these phenotypes and their regulation. For example, the Gac/Rsm and cyclic-di-GMP signaling networks both control expression of “acute” virulence determinants (e.g., type III secretion) and “chronic” virulence determinants (e.g., exopolysaccharides) [8], [9]. Yet the genetics and physiology of acute and chronic infections have not been directly compared *in vivo* using open-ended methods such as those enabled by recent advances in high-throughput genomics. Wounds represent an excellent system to study chronic and acute infections since both occur in soft tissues. Furthermore, mouse models of these infections recapitulate key features of infections in humans, such as rapid sepsis and mortality in acute infections, and prolonged healing times in both diabetic and non-diabetic chronic infections [10], [11].

In this study, we chose to study acute and chronic wound infections in mice using two complementary genomic technologies: RNA sequencing (RNA-seq) and transposon-junction sequencing (Tn-seq). Sequencing RNA-derived cDNA with high-throughput methods has proven to be a highly sensitive and comprehensive method for profiling bacterial transcriptomes [12]. Transcriptome-based approaches essentially use the infecting bacterium as a “biosensor”, using differential gene expression as a measure of the molecular and physiological cues sensed by the organism. Performing RNA-seq on RNA isolated from infected tissue from both model hosts and human patients has yielded genome-scale insights into differential gene expression by numerous bacteria during infection [13]–[15]. The requirement of individual genes for growth in an environment can be assessed genome-wide using Tn-seq. This method involves the quantitative sequencing of genomic DNA adjacent to a transposon insertion site to measure the abundance of an insertion mutant in a complex library containing tens or hundreds of thousands of individual mutants [16]–[18]. By subjecting this library to growth in a particular condition (such as infection of a model host) and subsequently profiling the abundance of each mutant by high-throughput sequencing, mutations that affect fitness in that condition can be identified upon comparison with an appropriate control condition. This approach has proven successful in identifying determinants of antibiotic resistance, carbon and energy utilization, and *in vivo* fitness [19]–[21]. However, despite the fundamentally distinct insights that can be gained by examining genome-wide gene expression and mutant fitness, few studies have performed these analyses on the same set of conditions, and none have done so during infection.

The goal of this work was to compare *P. aeruginosa* metabolism and virulence during models of acute and chronic infection. To this end, we performed RNA-seq and Tn-seq in *P. aeruginosa* grown *in vitro* and in two non-diabetic murine wound infection models, an acute infection model resulting in high levels of mortality and a non-lethal chronic infection model. Our results reveal that gene expression and mutant fitness are not correlated for most genes with the exception of metabolic genes, where differential expression is more predictive of a gene's role in fitness. By comparing gene expression and mutant fitness *in vivo* to growth in a defined medium, we reconstructed metabolism of *P. aeruginosa* during wound colonization and identified several metabolic pathways, including long-chain fatty acid catabolism, that are required for colonization and persistence. Additionally, we discovered that the ability to chemotax is required for *P. aeruginosa* fitness in burn but not chronic wound infections. These findings identify key features that are required for *P. aeruginosa* fitness in wound infections and demonstrate the utility of simultaneous gene expression and knockout fitness profiling for the study of bacterial metabolism, virulence, and physiology during infection.

## Results

### *P. aeruginosa* metabolic gene expression during infection

*P. aeruginosa* causes both acute and chronic wound infections, and we hypothesize that both bacterial and host factors mediate the outcome of wound infections. Here, we investigated *P. aeruginosa* wound infections from the perspective of the infecting bacterium to uncover similarities and differences between bacterial physiology in acute and chronic infections. Investigation of genetic requirements for *P. aeruginosa* colonization and persistence during infection requires animal models that encapsulate many of the key characteristics of human infections. In this study, two non-diabetic murine models of wound infection were used, one acute and one chronic. In the acute model, a dorsal full-thickness (third degree) burn is induced by scalding and infected subcutaneously with 10^2^–10^6^ *P. aeruginosa*. This infection is highly virulent, rapidly causing sepsis that leads to ∼100% mortality within 48 hours [11]. The chronic model involves infection of a surgically created full-thickness dorsal excision wound with 10^5^ *P. aeruginosa* that is covered by an adhesive dressing. This prevents contractile healing and ensures that these wounds heal by deposition of granulation tissue, much like human chronic wounds [22]. This infection can persist for weeks and is highly resistant to antibiotic treatment, and underlying conditions such as diabetes can extend the persistence time of these infections [10], [23]. Importantly for our purpose, these two infections can be initiated at approximately the same infecting dose with the same strain of *P. aeruginosa*.

To examine the physiology of *P. aeruginosa* during growth in these two model infections, we initially used RNA-seq (Table S1). The rationale for these experiments was that when compared to an appropriate control, transcriptomic methods such as RNA-seq can provide a genome-wide view of differential gene expression during infection, essentially using the infecting bacterium as a “biosensor” to report signals and cues sensed by the bacterium *in vivo*. As *P. aeruginosa* exhibits robust growth and persistence in these two infection models, much like in clinical infections, we were particularly interested in its primary metabolism during infection. Therefore, to interpret our RNA-seq results with a focus on central metabolism, we chose to compare the transcriptome of *P. aeruginosa* grown *in vivo* to growth in a defined minimal medium, specifically, growth to mid-logarithmic phase in a MOPS-buffered medium containing succinate as the sole carbon source (MOPS-succinate). This allowed comparison of metabolic gene expression in an unknown environment (*in vivo*) to an environment in which metabolism is largely understood (defined medium). We chose to profile the acute infection 40 hours post inoculation and the chronic infection 4 days post inoculation because these two timepoints represent midpoints of the trajectory of these respective infections. We reasoned that *P. aeruginosa* would have sufficient time to adapt to the infection environment and the host would have sufficient time to mount any immune response it was capable of raising at these timepoints. While no single timepoint can capture the dynamics of gene expression throughout the course of an infection, the timepoints chosen reflect a similar degree of progression in both wounds.

**Figure 1:**
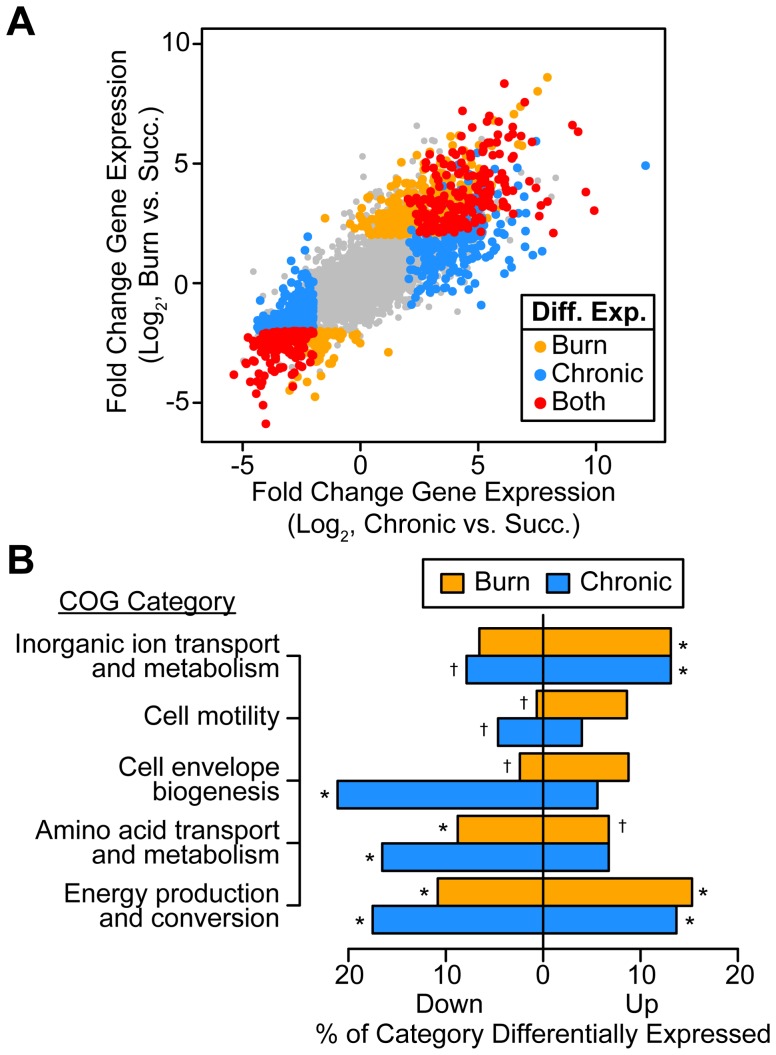
*P. aeruginosa* differentially regulates genes involved in metabolism, motility, and outer surface remodeling in wound infections. (A) Log_2_-transformed differential gene expression of *P. aeruginosa* in murine burn (y axis) and chronic (x axis) wound infections as compared to growth in MOPS-succinate (Succ.). Significant changes (fold change ≥4, P<0.01, negative binomial test) are colored as shown (Diff. Exp., differentially expressed in). (B) Significantly enriched or scarce COG categories in differentially expressed gene sets (P<0.01, Fisher's exact test; †, significantly less present than expected; *, significantly more present than expected).

We found that *P. aeruginosa* differentially regulates 14% and 19% of its genome during growth in murine burn and chronic wounds, respectively, as compared to MOPS-succinate (*P*<0.01, negative binomial test, fold change ≥4) (Table S2). The transcriptional responses of *P. aeruginosa* during growth in these two wound types as compared to MOPS-succinate are highly correlated (Spearman rank correlation coefficient = 0.840), suggesting that the cues sensed by *P. aeruginosa* in acute and chronic wound infections are largely similar (Figure 1A). Notably, 7.3% of the genome is commonly up- or down-regulated in both wound infections, which is a significant overlap (*P*<4.72×10^−110^, Fisher's exact test). The *P. aeruginosa* genome encodes numerous virulence factors, and our data provides a genome-wide perspective on the expression of these virulence genes (Table S3). We saw that many genes in the PA3160-PA3141 cluster, which encodes genes required for lipopolysaccharide O antigen biosynthesis [24], were down-regulated *in vivo*, and to a greater extent in chronic wounds. This suggests either that *P. aeruginosa* may alter its outer surface during infection, or that O antigen biosynthesis is regulated as a consequence of more static growth *in vivo*. We also saw that genes responsible for the biosynthesis of the siderophores pyochelin and pyoverdine were greatly up-regulated *in vivo*. Iron is known to be a limited resource in numerous infections, and our results suggest that iron acquisition is important in *P. aeruginosa* soft tissue infections as well [25]. Many type II and type III secretion system genes were up-regulated in both acute and chronic wounds as well, indicating that *P. aeruginosa* may be modulating host cellular physiology and extracellular environment through these well-characterized secretion systems [26], [27]. Finally, we saw down-regulation of many genes in the *psl* cluster, which is responsible for synthesis of the Psl exopolysaccharide. In strain PAO1, Psl is the primary exopolysaccharide involved in biofilm formation on abiotic surfaces [28]. Thus, *P. aeruginosa* differentially regulates much of its virulence repertoire upon wound infection, further underscoring the multifaceted nature of its virulence.

To determine what general features of *P. aeruginosa* physiology are altered *in vivo*, we performed COG enrichment analyses of genes differentially expressed in wounds as compared to MOPS-succinate (Figure 1B). As expected, genes involved in transport of inorganic ions, such as those encoding predicted ferric and ferrous iron transport systems, are enriched in the set of genes up-regulated *in vivo*. We also noted that amino acid biosynthetic genes are significantly enriched in the set of down-regulated genes in both wound types as compared to MOPS-succinate, suggesting that many amino acids are available in both chronic and acute wounds. Finally, the most extensive regulation *in vivo* was seen in COG category C, which includes genes involved in energy production and conversion, suggesting that the primary metabolism of *P. aeruginosa* is extensively remodeled during infection relative to growth in minimal media.

### Identifying determinants of *P. aeruginosa* fitness in wounds by Tn-seq

Our transcriptomic results suggest that bacterial gene expression is extensively regulated during infection. Yet it is unclear whether those genes that are differentially regulated play a role in *in vivo* fitness. To address this question, we chose to complement our *in vivo* transcriptomic studies with Tn-seq to identify the genetic determinants of bacterial fitness in acute and chronic wound infections. Briefly, a library of ∼100,000 *P. aeruginosa* transposon mutants [20] was grown in MOPS-succinate and in both acute and chronic wound models, and mutant abundance was profiled by Tn-seq either 24 hours or 3 days post inoculation for the acute and chronic infections, respectively (Table S4). As the abundance of a particular mutant in the library will be influenced by its relative fitness throughout the history of the library, these timepoints are sufficient to query genes required for both initial colonization and subsequent growth in these infections. We found that 11% and 16% of the genome contributes to fitness in murine burn and chronic wounds as compared to growth in MOPS-succinate (*P*<0.05, negative binomial test, fold change ≥4), respectively, and that 3% of the genome contributes to fitness in both wounds, which is a significant overlap (*P*<1.66×10^−25^, Fisher's exact test) (Table S5). We first examined the fitness contribution of known virulence factors (Table S6). We saw that the flagellum is required only in burn wounds, confirming previous studies and the validity of our Tn-seq approach [29]. Many genes in the type III and the type VI secretion systems contribute to fitness in chronic wounds, further suggesting that inter-cellular delivery of effector proteins may be important in these wounds. Interestingly, despite their down-regulation, the *psl* exopolysaccharide genes contribute to fitness in both acute and chronic wounds. Finally, some genes involved in producing type IV pili, another motility system [30], appear to be required in both acute and chronic wounds. Taken together, our results emphasize that virulence in *P. aeruginosa* is multifactorial, involving the coordinated action of motility, biofilm formation, and secretion systems.

### Genome-wide gene expression and knockout fitness are not generally correlated

**Figure 2:**
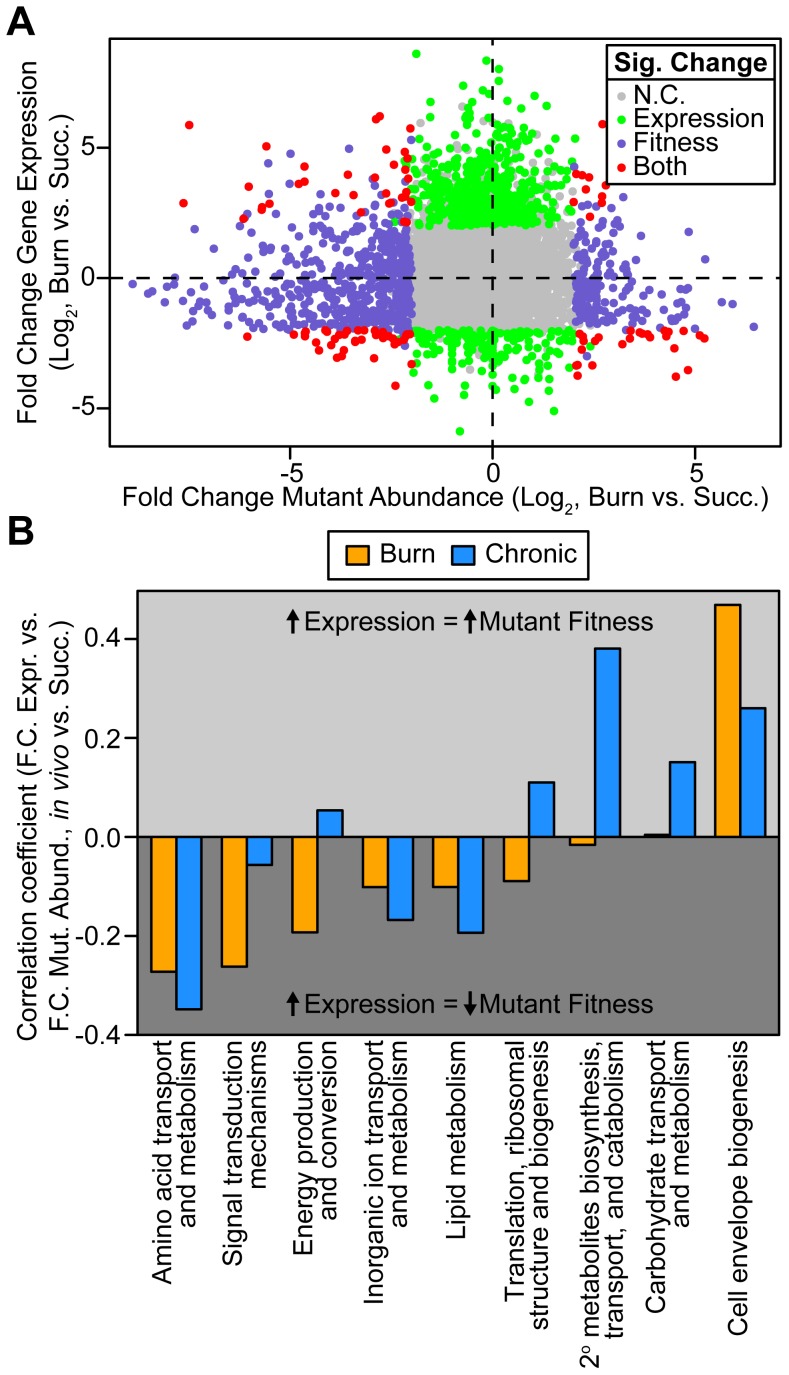
Genome-wide *P. aeruginosa* gene expression and knockout fitness in wound infection are not correlated. (A) Log_2_-transformed fold change gene expression (y axis) and knockout abundance (x axis) of *P. aeruginosa* in murine burn wound infections as compared to growth in MOPS-succinate (Succ.). Significant (Sig.) changes in gene expression (fold change ≥4, P<0.01, negative binomial test) and mutant abundance (fold change ≥4, P<0.05, negative binomial test) are colored as shown (N.C., no change). (B) Spearman rank correlation coefficient between fold change expression and fold change mutant abundance in the burn wound-MOPS-succinate and the chronic wound-MOPS-succinate comparisons (x axis) for COG categories with more than 10 differentially regulated members and an associated correlation greater than 0.1 or less than −0.1 (y axis). Only genes with transposon-derived Tn-seq reads were considered.

Since transcriptomics has been used in the past to identify bacterial genes potentially important for *in vivo* fitness [14], [15], [31], we hypothesized that genes identified as important for fitness using Tn-seq would display increased expression *in vivo*. If this hypothesis is true, one would expect that a correlation coefficient (which expresses correlation between two variables on a scale from −1, or perfectly anticorrelated, to 1, or perfectly correlated) would be closer to −1. However, we found that mutant fitness and differential expression are uncorrelated, suggesting that in this case RNA-seq is not a good predictor of genes important for fitness in wounds (Figures 2A and S1, Table 1). One should keep in mind that Tn-seq is a competitive infection since most strains are wild-type for a given genetic locus, and RNA-seq may be more predictive if individual mutants are examined.

**Table 1:** Differential expression and mutant fitness are poorly correlated.

Genes considered	ρ^1^, Burn vs. Succ.	ρ, Chronic vs. Succ.	ρ, Chronic vs. Burn
All with Tn-seq reads	0.051 (5,296^2^)	0.006 (5,265)	−0.028 (5,115)
DE^3^, with Tn-seq reads	0.011 (740)	0.068 (906)	0.200 (148)
DE, with Tn-seq reads and unique EC^4^ number	0.013 (80)	−0.070 (130)	0.217 (12)

1 Spearman rank correlation coefficient between fold change expression and fold change mutant abundance. More negative indicates a greater fitness contribution by up-regulated genes and/or a lesser fitness contribution by down-regulated genes.

2 Number of genes considered is shown in parentheses.

3 Differentially expressed (fold change ≥4, P<0.01, negative binomial test).

4 Enzyme Commission.

Although it is clear that Tn-seq and RNA-seq results are not correlated when all genes are examined, we hypothesized that particular subsets of genes may show a stronger correlation. If this is the case, identifying these subsets of genes would have the potential to guide hypotheses regarding genes important for fitness in bacteria with poor genetic tools, or in natural microbial populations such as those associated with primary human samples, where methods like Tn-seq are not feasible. One possible subset of genes that may be more predictive are those that are most highly differentially regulated. To test this, genes were ranked from high to low fold-change expression and correlated with fitness scores for ever-increasing subsets of genes along that ranking. No significant improvement in correlation was observed, indicating that the magnitude of differential *in vivo* expression is not more predictive of fitness (Figure S2). We found that the same is true for genes that contributed strongly to fitness, as ranking from low to high mutant fitness also does not enhance the correlation. As Tn-seq measures the fitness of single mutants, we hypothesized that genetic redundancy might mask a role of some genes in fitness, and that limiting our analysis to genes without predicted redundancy might improve expression-fitness correlation. To test this, Enzyme Commission (EC) numbers, which describe the enzymatic function of a gene product, were used to determine which genes lack functional paralogs elsewhere in the genome. However, limiting our analysis to those differentially expressed genes with a unique EC number did not substantially alter expression-fitness correlation (Table 1). It should be noted that this approach does not address more complex manifestations of redundancy, such as robustness in functional gene interaction networks [32], which may contribute to the lack of correlation between our Tn-seq and RNA-seq results.

### Differential expression predicts fitness contribution better for metabolic genes

We next examined whether the predictive power of gene expression for mutant fitness is better for certain functional classes of genes. Therefore, we examined expression-fitness correlation by COG category. We saw that differential expression and mutant fitness are more negatively correlated in both wound models for several COG categories (Figure 2B). One of these COG categories is amino acid metabolism and transport, suggesting that, relative to growth in MOPS-succinate, *P. aeruginosa* down-regulates amino acid biosynthetic genes *in vivo* to avoid the fitness cost associated with expressing them when they are not needed (Figure 2B). We also saw that differential expression and mutant abundance are more negatively correlated for genes in the energy production and conversion, lipid metabolism, and inorganic ion transport and metabolism COG categories. For genes in these categories, up-regulation is more predictive of a fitness defect of mutants lacking those genes (Figure 2B), suggesting that changes in metabolic gene expression are adaptive, conferring a fitness benefit on the organism. These results underscore the importance of scavenging available nutrients and limited-availability ions (such as amino acids and iron) while up-regulating key central metabolic pathways during infection.

### A reconstruction of primary metabolism *in vivo* based on transcriptomic data

**Figure 3:**
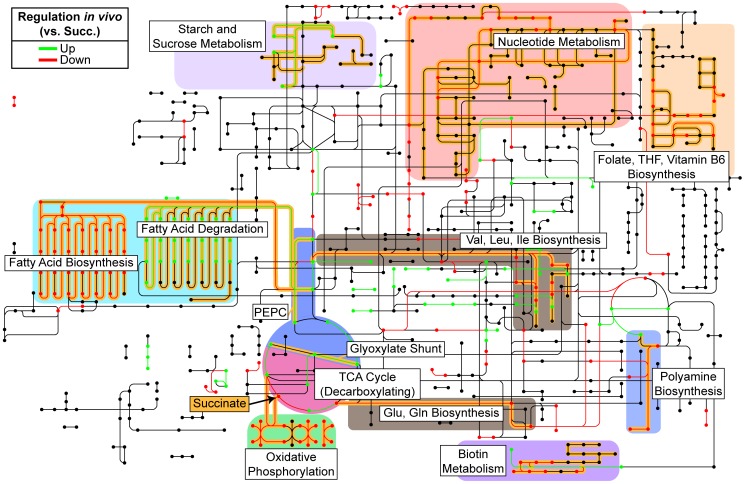
Reconstruction of *in vivo* metabolism from RNA-seq data. Metabolic steps as identified by KEGG Orthology are colored according to their relative expression in both burn and chronic wounds as compared to growth in MOPS-succinate (Succ.). Only significant changes (fold change ≥4, P<0.01, negative binomial test) in both burn and chronic wounds are shown. Pathways and metabolic steps of interest are highlighted in yellow, and succinate, the sole carbon and energy source in the control medium, is indicated with an arrow. Generally, biosynthetic pathways were down-regulated and anaplerotic pathways were up-regulated *in vivo*. (PEPC, PEP carboxylase; THF; tetrahydrofolate).

Our analysis of the correlation between differential expression and conditional mutant fitness by COG category (Figure 2B) indicates that expression is a better predictor of fitness contribution for genes involved in primary metabolism. Therefore, to characterize the primary metabolism of *P. aeruginosa* during wound infection, we projected our transcriptome profiling results onto the Kyoto Encyclopedia of Genes and Genomes (KEGG) PATHWAYS database (Figure 3). As mentioned previously, our choice of a defined medium (MOPS-succinate) as a control condition provided a reference point from which to understand bacterial physiology and metabolism in the unknown nutritional environment of the infected wound. Our metabolic reconstruction suggests that genes encoding decarboxylating steps of the TCA cycle (isocitrate dehydrogenase and α-ketoglutarate dehydrogenase) are down-regulated, and that the gene encoding the entry point to the glyoxylate shunt (isocitrate lyase) is up-regulated. The glyoxylate shunt is a variation on the TCA cycle not present in mammals that allows bacteria, including *P. aeruginosa*, to grow on reduced carbon sources such as fatty acids by bypassing TCA cycle reactions that would result in the loss of carbon [33]. This serves to replenish TCA cycle intermediates utilized in biosynthesis, which is known generally as anaplerosis. We also observed up-regulation of *ppc*, which encodes a second non-mammalian anaplerotic enzyme, PEP carboxylase, in wound infections, though it is much more highly expressed in chronic wounds than in acute wounds (Table S2). Finally, expression of a number of genes associated with oxygen-limited environments is also up-regulated, including those encoding high-affinity terminal oxidases and the denitrification pathway [34], suggesting that at least some bacterial cells in these infections sense decreased oxygen tension. This is consistent with frequent observations of ischemia at wound sites in the clinic, and suggests that oxygen limitation affects the physiology of both the infecting organism and host tissue at wound sites [35]. The transcriptomic results suggest that *P. aeruginosa* differentially regulates a large portion of its genome in soft tissue infections, and that this reflects a response to differential availability of key metabolic factors such as carbon and energy sources, biosynthetic endproducts, and terminal electron acceptors. In the remainder of this manuscript, we will examine each of these in detail.

### Long-chain fatty acids are an energy source used by *P. aeruginosa* in wounds

**Figure 4:**
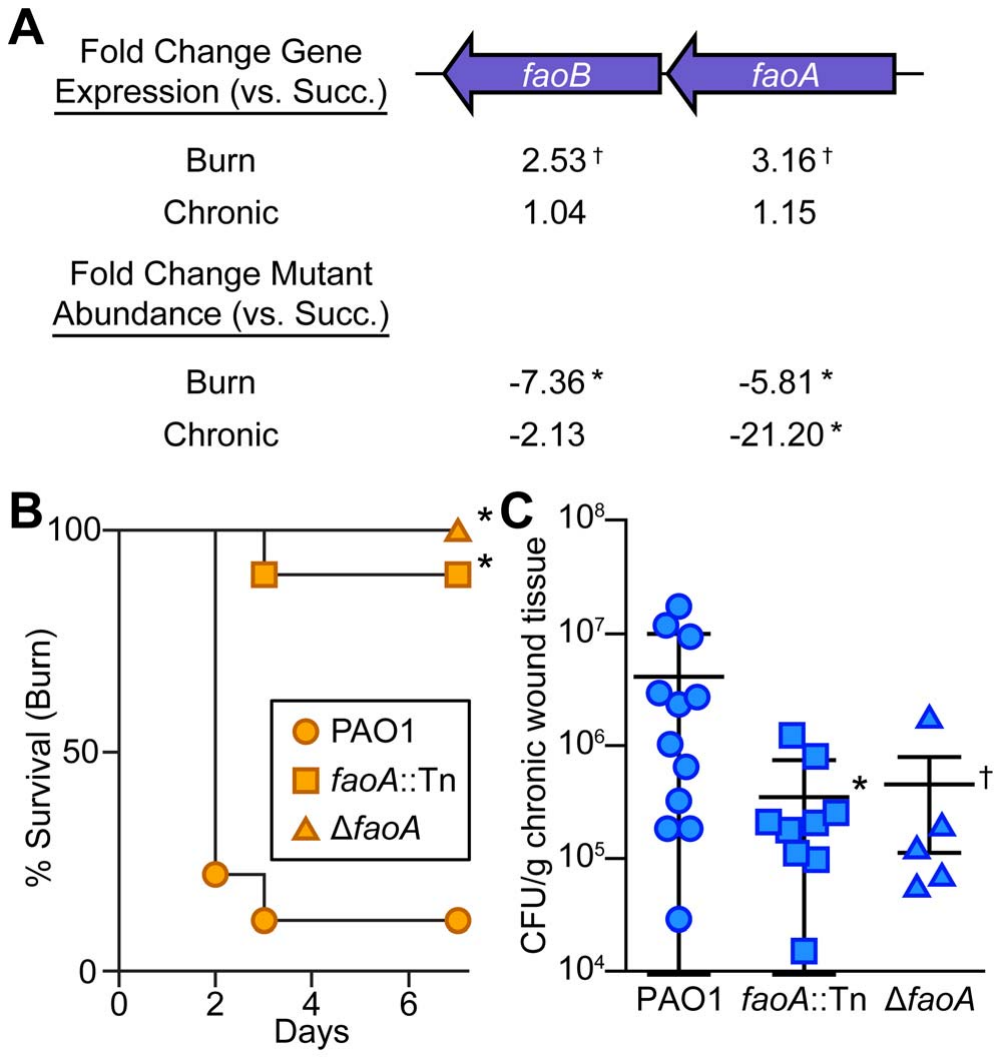
Long-chain fatty acid oxidation is required for *P. aeruginosa* virulence and fitness in wounds. (A) Fold change gene expression (above) or mutant abundance (below) in murine wound infections as compared to MOPS-succinate (Succ.) is shown for the long-chain fatty acid oxidation genes *faoAB* (*, P<0.05, negative binomial test; †, P<0.01, negative binomial test) (B) Kaplan-Meier survival curves of burned mice infected with wild-type PAO1, an *faoA* transposon mutant derivative (*faoA*::Tn), or an unmarked, in-frame *faoA* deletion mutant (Δ*faoA*). The experiment was performed twice (PAO1, *faoA*::Tn) or once (Δ*faoA*), with three to five mice per group, and the percent survival of all mice is shown (*, P<0.005, log-rank (Mantel-Cox) test; n = 8 (PAO1), n = 10 (*faoA*::Tn), n = 5 (Δ*faoA*)). (C) Growth of wild-type PAO1, *faoA*::Tn, or Δ*faoA* in murine chronic wounds four days post infection. Each symbol represents a value obtained from infection of an individual mouse. The central bar indicates the mean, and error bars indicate standard error of the mean (*, P = 0.023, unpaired T-test; †, P = 0.091, unpaired T-test).

Infected host tissue is a complex nutritional environment for a bacterium, with many potential metabolites available for bacterial catabolism. Manipulation of key metabolites during infection has therapeutic potential in much the same way as arginine-auxotrophic cancers can be treated by depletion of available L-arginine [36]; however, the nutrients utilized by bacteria in wound infections are not known. Comparing the expression of primary metabolic genes *in vivo* to growth in defined minimal media led us to hypothesize that fatty acids are a primary carbon source available to *P. aeruginosa in vivo* (Figure 3). Examination of our Tn-seq data in detail (Table S4) revealed that the *faoAB* (or *fadBA5*) genes, which are required for robust growth on long-chain (C12 or greater) fatty acids [37], contribute to *P. aeruginosa* fitness *in vivo* (Figures 4A and S3A). This was confirmed by single mutant infections: both an *faoA* transposon mutant and an *faoA* deletion mutant are attenuated in both acute and chronic wounds, indicating that long-chain fatty acids are likely an important energy source in wounds (Figure 4BC). The *faoAB* genes have also been shown to contribute to resistance to tobramycin, so they could potentially contribute to resistance to an unspecified chemical stress *in vivo* as well [20]. However, our *in vivo* gene expression data suggests that growth in wound infections involves pathways active during growth on reduced carbon sources such as long-chain fatty acids (Figure 3). We did not observe an *in vivo* fitness defect for genes annotated as homologs of the *Escherichia coli* long-chain fatty acid outer membrane transporter gene *fadL* (*fadL1*, *fadL2*, or *fadL3*) in *P. aeruginosa*; however, these genes are not thought to be required for long-chain fatty acid transport in *P. aeruginosa* [38].

### Tn-seq reveals metabolites available to *P. aeruginosa in vivo*

**Figure 5:**
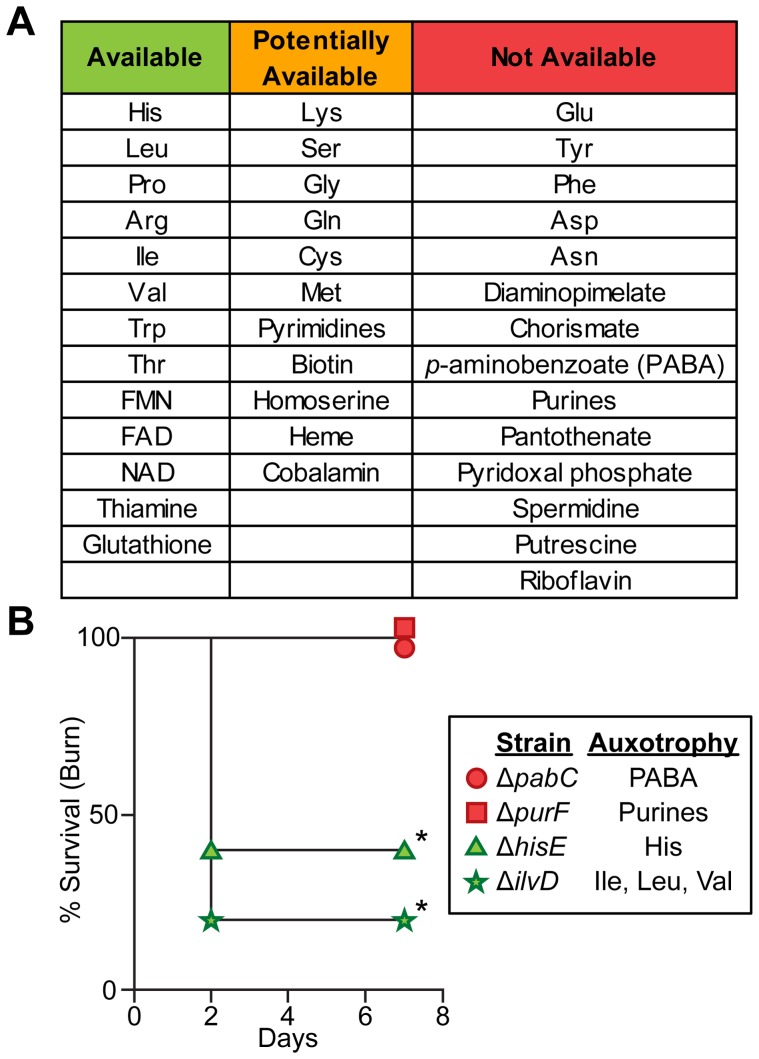
Biosynthetic requirements of *P. aeruginosa* during wound infection. (A) *P. aeruginosa* must biosynthesize several amino acids, cofactors, and metabolic endproducts during growth *in vivo*. If mutants in >33% of genes predicted to be in the biosynthetic pathway of a given metabolite were more fit in both burn and chronic wounds than in MOPS-Succ (fold change ≥2, P<0.05, negative binomial test), that metabolite was said to be “Available” to *P. aeruginosa in vivo*. If 5–33% of genes predicted to be in the biosynthetic pathway of a given metabolite were more fit in both burn and chronic wounds than in MOPS-Succ, or if only one infection matched the criteria for “Available” as described above, that metabolite was said to be “Potentially Available”. If <5% of genes predicted to be in the biosynthetic pathway of a given metabolite were more fit in both burn and chronic wounds than in MOPS-Succ, that metabolite was said to be “Not Available”. Genes predicted to be involved in synthesis of necessary metabolites were identified from the PseudoCyc database (see Table S7 for details). (B) Kaplan-Meier survival curves of burned mice infected with PAO1 Δ*pabC*, PAO1 Δ*purF*, PAO1 Δ*hisE*, or PAO1 Δ*ilvD*. The first two strains are auxotrophic for metabolites predicted to be not available, and the last two strains are auxotrophic for metabolites predicted to be available. The experiment was performed with five mice per group, and the percent survival of all mice is shown (*, P<0.05, log-rank (Mantel-Cox) test).

In addition to identifying primary carbon and energy sources during wound infections, our results also allow identification of biosynthetic end products that are available to *P. aeruginosa* in wound infections. We reasoned that biosynthetic pathways required in minimal media but dispensable *in vivo* would likely be responsible for the synthesis of metabolites available to *P. aeruginosa in vivo*. To identify biosynthetic genes, we used the manually curated PseudoCyc annotation [39]. Metabolites for which 33% or more of the biosynthetic genes contribute more to fitness in MOPS-succinate than in both acute and chronic wounds (*P*<0.05, negative binomial test, fold change ≥2) were deemed “available”, and include many amino acids, the electron carriers FAD and NAD, and the B vitamin thiamine (Figure 5A and Table S7). Metabolites for which 95% or more of the biosynthetic genes have a similar effect on fitness in MOPS-succinate and in both acute and chronic wounds were deemed “not available”, and include the amino acids glutamate, tyrosine, phenylalanine, aspartate, and asparagine, purines, many other vitamins and cofactors including the folate precursor *p*-aminobenzoate (PABA) and several other B group vitamins. The remaining metabolites that do not match either of the above sets of criteria were deemed “potentially available”. To confirm the validity of this approach, we constructed two in-frame, unmarked deletion mutants lacking the ability to biosynthesize metabolites predicted to be unavailable in wounds: one lacking *pabC* (which requires PABA for growth in a minimal medium) and one lacking *purF* (which requires purines for growth in a minimal medium) (Figure S3B). These two mutants were completely attenuated for virulence in the burn wound (Figure 5B). However, in-frame, unmarked deletion mutants unable to grow without histidine or without isoleucine, leucine, and valine, all of which are predicted to be available in wounds, were significantly more virulent than the *pabC* or *purF* mutants. Thus, by comparison with minimal media, we demonstrate that genome-wide bacterial mutant fitness can be used to comprehensively profile bioavailable metabolites in a complex, undefined environment. Bacterial-specific pathways responsible for biosynthesis of any of the unavailable metabolites identified may represent promising targets for therapeutic intervention in wounds.

### Chemotaxis is required in acute but not chronic wound infections

**Figure 6:**
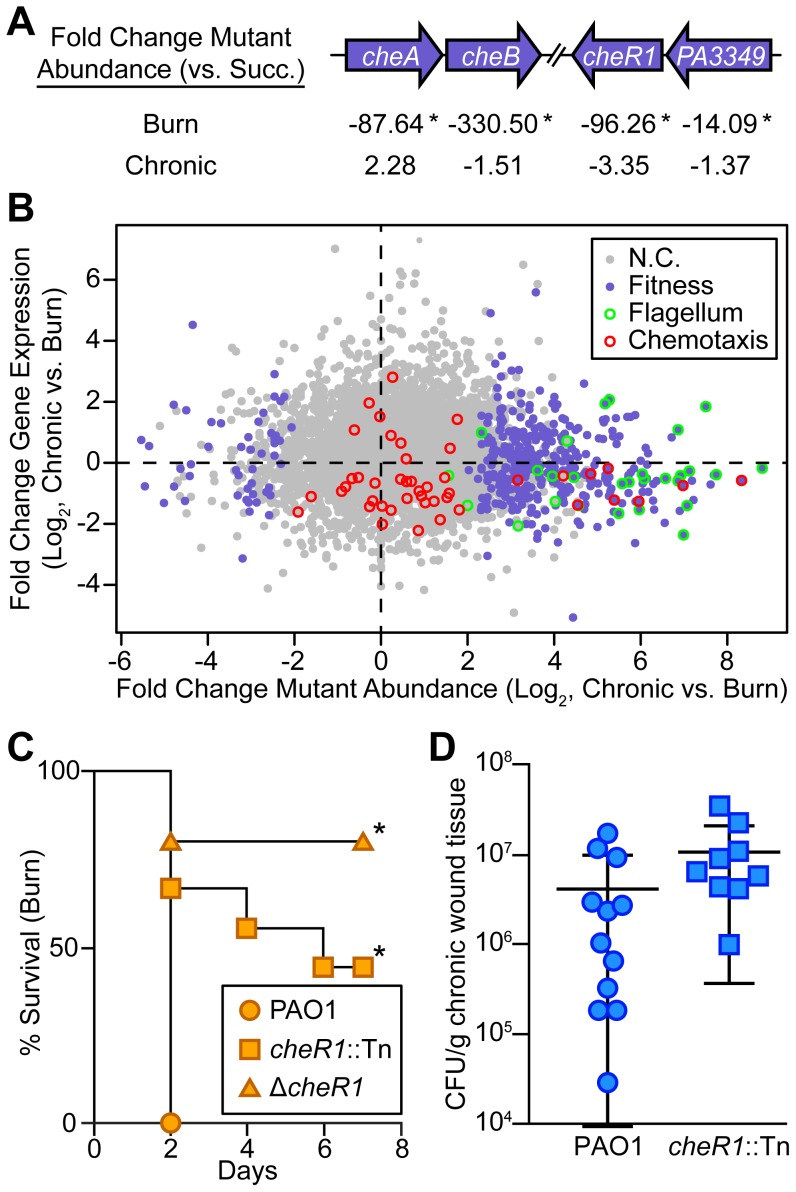
Chemotactic flagellar motility is required in burn wounds and not chronic wounds. (A) Fold change mutant abundance in murine wound infections as compared to MOPS-succinate (Succ.) is shown for genes involved in chemotaxis (*, P<0.05, negative binomial test). (B) Log_2_-transformed fold change gene expression (y axis) and knockout abundance (x axis) of *P. aeruginosa* in murine chronic wound infections as compared to murine burn wound infections. Significant changes in knockout abundance (fold change ≥4, P<0.05, negative binomial test) are colored purple, and genes annotated as being involved in flagellar assembly or bacterial chemotaxis in the KEGG PATHWAYS database are highlighted green and red, respectively (N.C., no change). (C) Kaplan-Meier survival curves of burned mice infected with wild-type PAO1, a *cheR1* transposon mutant derivative (*cheR1*::Tn), or an unmarked, in-frame *cheR1* deletion mutant (Δ*cheR1*). The experiment was performed twice (PAO1, *cheR1*::Tn) or once (Δ*cheR1*), with three to five mice per group, and the percent survival of all mice is shown (*, P<0.01, log-rank (Mantel-Cox) test; n = 8 (PAO1), n = 9 (*cheR1*::Tn), n = 5 (Δ*cheR1*)). (D) Growth of wild-type PAO1 or a *cheR1* transposon mutant derivative in murine chronic wounds four days post infection. Each symbol represents a value obtained from infection of an individual mouse. The central bar indicates the mean, and error bars indicate standard error of the mean. No significant difference was observed (P = 0.194, unpaired T-test).

While our focus on metabolism revealed numerous similarities in chronic and acute infections, the genomic techniques employed here also provided new insight into how these infection types differ (Table S8). As a motile bacterium, *P. aeruginosa* possesses the ability to detect and move toward nutrients (including long-chain fatty acids [38]), a process referred to as chemotaxis. Examination of our Tn-seq results revealed that several genes with putative roles in chemotaxis, including *cheA*, *cheB*, *cheR1*, and a homolog of *cheW* (PA3349) are required in burn, but not chronic wounds (Figure 6A). In addition nearly every annotated flagellar gene is required for fitness in burn wounds [29], but is dispensable in chronic wounds (Figure 6B). To further confirm the role of chemotaxis in acute wound infections, single-strain infections with a *cheR1* transposon insertion mutant and an in-frame, unmarked *cheR1* deletion mutant (Figure S3C) were performed. While both the *cheR1* insertion and deletion mutants have virulence defects in burn wounds (Figure 6C), the *cheR1* insertion mutant is as fit or more fit than wild-type *P. aeruginosa* in chronic wounds (Figure 6D). These results suggest that the ability to chemotax along a spatio-chemical gradient by utilizing flagellar motility is a key feature of acute but not chronic wound *P. aeruginosa* infections.

## Discussion

The opportunistic pathogen *P. aeruginosa* is remarkably versatile, able to thrive in a wide range of environments and cause infections in diverse tissue types. These infections can have wide-ranging timescales, from mere days in acute infections such as those in burn wounds or in the cornea to the decades-long pulmonary infections associated with cystic fibrosis [1]. Remarkably, *P. aeruginosa* is able to achieve this breadth of infectious capability with highly conserved genomic content [40]. This suggests that the capacity to be an acute or a chronic pathogen is innate to the organism, and is determined largely by the context in which the infection is found. Using two complementary genomic techniques, we have investigated the physiology and fitness of *P. aeruginosa* in two soft tissue infections, one acute and one chronic, and shown similarities and differences between them. Interestingly, with the exception of chemotactic motility, there do not seem to be many infection type-specific genetic pathways required for fitness in one infection versus the other, suggesting that components of host physiology, likely the immune system, dictate the fate of soft tissue infections.

While our data include numerous implications for the role of characterized virulence systems in wound infections, we have chosen to focus mainly on metabolic genes in this study. The reason for this is three-fold: (1) We found that, when compared to growth in a defined medium, metabolic gene expression could be easily interpreted and correlated well with mutant fitness. This approach allowed us to fully profile catabolism, anabolism, and respiration for an infecting bacterium solely from transcriptomic data, which may prove useful in the study of bacterial physiology in conditions in which mutant fitness experiments are not feasible (such as human infections). (2) Bacterial metabolism during infection is poorly understood. The primary carbon and energy sources utilized by bacteria during infection are only known for a few instances [41]. Our data suggest that by comparing Tn-seq data obtained after growth *in vivo* to that obtained in a defined minimal medium can make great inroads towards a greater understanding of metabolism during infection. (3) Modulation of the host metabolic environment has shown promise in treating other diseases characterized by fast-growing and invasive cells such as cancer [36], and has immediate therapeutic potential for treatment of infections. Our data suggest that interfering with long-chain fatty acid catabolism (Figure 4) or transport, or biosynthesis of several key metabolites (Figure 5) in *P. aeruginosa* wound infections may impair bacterial fitness *in vivo*.

As in other Gram-negative bacteria, the core chemotaxis system of *P. aeruginosa* transduces signals from methyl-accepting chemotaxis proteins (MCPs), each of which is thought to respond to a distinct signal, to the flagellar motor, ultimately resulting in chemotaxis along a gradient [42]. We found by Tn-seq that the major aerotaxis receptor gene *aer* is a fitness determinant in burn wounds, suggesting a role for aerotaxis in burn wounds (Tables S5 and S8). However, the *P. aeruginosa* genome encodes at least two aerotaxis MCPs [43], and mutants lacking either *aer* alone or *aer* and *aer2* together exhibited full virulence in single-strain burn wound infections (data not shown), suggesting that multiple MCPs can contribute to chemotaxis in acute wounds. Thus, genetic redundancy may mask the role of other genes in fitness in studies of single mutant strains such as ours. This weakness may be exacerbated in bacteria with large genomes that contain more paralogs, like *P. aeruginosa* [44]. In addition to the function of paralogs, genetic redundancy may also result from robustness in genetic interaction networks, which is more difficult to predict *a priori* [32]. The relative lack of chronic wound-specific single mutant phenotypes (Figure 6B) may be partially attributable to this redundancy. Therefore, systematic approaches to examine the phenotypes of double and triple mutants are needed to uncover the basis for and importance of polygenic traits in bacteria.

A second weakness inherent to pooled selection approaches such as Tn-seq is cross-complementation, in which the lack of a particular gene product in one strain is complemented by the production of that product by a neighboring strain. This is often thought of in the context of “public goods”, which are often equated with secreted products [45]. However, we noted that several genes presumed to be involved in the production of secreted products, such as the siderophores pyochelin and pyoverdine, are required of an individual strain in co-infection (Table S6). This suggests that some secreted products may confer a benefit on the secreting cell without fully transferring those benefits to neighboring cells. The extent to which cross-complementation affects phenotypes of other mutants lacking certain products thought to be cytoplasmic is unclear as well. For example, are metabolic precursors shared between strains, and does that affect our ability to identify essential anabolic pathways by Tn-seq (Figure 5)? Further study on the exact nature and molecular basis of public goods is required, and can help inform our understanding of community interactions both within and between species during infection.

By investigating the correlation between gene regulation and knockout fitness, we showed that, generally, *P. aeruginosa* gene regulation in wound infections is nonadaptive. As an opportunistic pathogen whose evolutionary trajectory is not thought to be shaped by its fitness in mammalian infections, it is not surprising that regulation of factors that contribute to fitness in wounds is not necessarily tied to signals or cues present in the infection environment. In longer-lasting infections where *P. aeruginosa* can evolve to be more fit in its environment, such as in the cystic fibrosis lung, adaptive changes in global gene expression have been observed over time [46]. This suggests that *P. aeruginosa* gene regulation can be better “tuned” by evolution to express *in vivo* fitness determinants. It would be interesting to explore expression-fitness correlation in “professional” pathogens, as it may be improved in organisms that are more adapted for growth in the human host.

## Materials and Methods

### Ethics statement

This study was carried out in strict accordance with the recommendations in the Guide for the Care and Use of Laboratory Animals of the National Institutes of Health. The protocol was approved by the Institutional Animal Care and Use Committee of Texas Tech University Health Sciences Center (Protocol Numbers 07044 and 96020).

### Bacterial strains and growth media

*P. aeruginosa* PAO1 transposon insertion mutants, including the ∼100,000 transposon mutant library and individual transposon mutants in *faoA* (strain PW6048) and *cheR1* (strain PW6640), were obtained from Colin Manoil (University of Washington) [20], [47]. Transposon insertions were confirmed by PCR. For single-strain infection experiments, the parental PAO1 strain was used. The PAO1 strain used in RNA-seq experiments was obtained from Dennis Ohman (Virginia Commonwealth University). Growth *in vitro* was performed either in LB Miller broth, in morpholine-propanesulfonic acid (MOPS)-buffered minimal medium [48] with 20 mM succinate (hereafter referred to as MOPS-succinate), or in MOPS-buffered minimal medium with 0.2% oleic acid and 1% Brij-58 [37] (hereafter referred to as MOPS-oleate) shaking at 250 rpm at 37°C. *In vitro* cultures for Tn-seq analysis were grown as follows: frozen aliquots of the PAO1 transposon insertion library were washed twice with 1 mL MOPS-buffered media base, inoculated into 10 mL media at 10^6^ CFU/mL and grown for approximately 9 generations (to ∼10^9^ CFU/mL). *In vitro* cultures for RNA-seq analysis were grown overnight in the test media, diluted to an OD600 of 0.01, and grown to mid-logarithmic phase (OD600 = ∼0.5) before harvesting as described below.

### Construction and testing of deletion mutants and complementation plasmids

Deletion constructs contain two 600–800 basepair (bp) fragments flanking the gene of interest in which the coding sequence of the gene of interest was replaced by the sequence 5′-GCGGCCGCC-3′ (preserving the native start and stop codons). This insert was cloned into plasmid pEXG2 [49] on a SacI-KpnI fragment, and these deletion alleles were introduced to strain PAO1 by allelic exchange to generate strains PAO1 Δ*purF*, PAO1 Δ*pabC*, PAO1 Δ*ilvD*, PAO1 Δ*hisE*, PAO1 Δ*faoA*, and PAO1 Δ*cheR1* as described [50]. Complementation plasmids were constructed by amplifying the coding sequence of the gene of interest (including the native start and stop codons) by PCR with Phusion Hot Start II DNA Polymerase (Thermo Scientific, Waltham, MA). The forward primer in these reactions had a 5′ tail of 5′-GCTATGACCATGATTACGAATTCNNNNNNNNTACAT-3′, and the reverse primer in these reactions had a 5′ tail of 5′-CATGCCTGCAGGTCGACTCTAGA-3′. PCR products and the plasmid pUCP18 (linearized by triple digestion with SacI, BamHI, and KpnI) were gel purified, and the PCR product was introduced to the plasmid backbone by Gibson assembly as described [51]. This generated a library of >300 plasmids in *E. coli* strain DH5a for each plasmid, and these plasmid libraries were prepared from *E. coli*. Then, these plasmid libraries were used to transform the appropriate mutant PAO1 derivative to be complemented by electroporation, and complementing plasmids were isolated as follows: (1) For plasmids pPurF and pPabC, the pPurF and pPabC candidate plasmid libraries were transformed into PAO1 Δ*purF* and PAO1 Δ*pabC*, respectively, and complemented electroporants were isolated by plating on solid MOPS-succinate agar and restreaking colonies on solid MOPS-succinate agar for isolation. (2) For plasmid pFaoA, the pFaoA candidate plasmid library was transformed into PAO1 Δ*faoA*, and complemented electroporants were isolated by plating on solid MOPS-oleate agar and restreaking colonies on solid MOPS-oleate agar for isolation. (3) For plasmid pCheR1, the pCheR1 candidate plasmid library was transformed into PAO1 Δ*cheR1*, and complemented electroporants were isolated by spotting the transformation mix on semisolid LB media containing 0.3% agar supplemented with 150 µg/mL carbenicillin and restreaking a region that had swam out from the initial spot for isolation on solid LB agar supplemented with 150 µg/mL carbenicillin. At least three individual complemented strains were tested as shown in Figure S3 for each deletion, with a representative strain shown for each. For the burn wound infections shown in Figure 5B, mid-logarithmic phase cells of the indicated PAO1 mutants were starved for 2 hours in MOPS buffer before inoculation as described below.

### Murine burn wound infections

Murine burn wound infections were performed with adult female Swiss Webster mice essentially as described [11], with the following modifications. For Tn-seq experiments, 10^6^ CFU of the PAO1 transposon mutant library was used as an inoculum, and wound tissue was harvested 24 hours post infection and stored in RNAlater (Qiagen) at room temperature for 24–48 hours, and subsequently at −20°C. For RNA-seq experiments, 10^5^ CFU of wild-type PAO1 was used as an inoculum, and wound tissue was harvested 40 hours post infection as described above. For single strain infections, 10^2^–10^3^ CFU of the indicated strain was used as an inoculum, and animals were monitored for mortality daily for up to 7 days. Each experiment was performed at least twice with at least 5 animals per experimental group, and the average time of death for all animals is reported here.

### Murine chronic wound infections

Murine chronic wound infections were performed with non-diabetic adult female Swiss Webster mice essentially as described [23], with the following modifications. For Tn-seq experiments, 10^5^ CFU of the PAO1 transposon mutant library was used as an inoculum, and wound tissue was harvested 3 days post infection and stored in RNAlater as described above. For RNA-seq experiments, 10^5^ CFU of wild-type PAO1 was used as an inoculum, and wound tissue was harvested 4 days post infection as described above. For single strain infections, 10^5^ CFU of the indicated strain was used as an inoculum, wound tissue was harvested 4 days post infection, and CFU/g tissue was determined by plating. Each experiment was performed at least twice with at least 5 animals per experimental group.

### Tn-seq Illumina library preparation

To prepare DNA for Tn-seq analysis, ∼100 mg sections of wound tissue or cell pellets were resuspended in 1 mL 1× Buffer A [52] +0.1% SDS, homogenized in a Mini-Beadbeater (Biospec) in 2 mL vials preloaded with Lysing Matrix B (MP Biomedicals) 3–5 times for 1 minute each, resting on ice in between each pulse. Proteinase K was then added to 1 mg/mL, and samples were incubated overnight at 50°C. Samples were then homogenized once more as above, separate sections from the same wound were pooled, and samples were extracted with an equal volume of 25∶24∶1 phenol∶chloroform∶isoamyl alcohol pH 8.0. DNA was ethanol precipitated from the aqueous phase, and was resuspended in 200–500 µL water after extensive pellet washing with 75% ethanol. Tn-seq sequencing libraries were prepared by a modified version of INSeq [16]. DNA was sheared to approximately 500 bp either in a S220 Focused-ultrasonicator (Covaris), a Hydroshear Sonicator (Digilab), or a Q880R Sonicator (Qsonica). 500 ng (*in vitro* samples) to 1 µg (murine wound samples) of DNA was used as template in two linear PCR reactions using the 5′ biotinylated oligonucleotide primer Kbio-T8OE-Out2 (5′-ATAAGAATGCGGCCGCGGGATGGAAAACGGGAAAGGTTCCGTCCAGGACGCTACTTGTG-3′) and performed with KOD Hot Start DNA Polymerase (EMD Biosciences) with the following program: 95°C, 5′; 99×(95°C, 30″; 68°C, 1′); 68°C, 10′. Kbio-T8OE-Out2 is specific to the “OE” end of transposon T8 [47], with two key features: (1) A NotI site is contained towards the 5′ end of the primer for NotI cleavage-mediated elution (see below), and (2) the primer sequence ends 12 bp from the end of transposon T8, leaving that additional 12 bp sequence for additional sequence quality control. Biotinylated linear PCR products were bound to Streptavidin-coupled Dynabeads (Invitrogen) and a second strand was synthesized as described [52], except that the oligonucleotide used to prime second strand synthesis had the sequence 5′-NSNSNSNSNS-3′. Double-stranded DNA was eluted from the Dynabeads by digesting with NotI-HF (New England Biolabs), and this DNA was prepared for Illumina sequencing with the NEBNext DNA Library Prep Master Mix Set For Illumina (New England Biolabs) according to the manufacturer's protocol. Libraries were sequenced at the Genome Sequencing and Analysis Facility at the University of Texas at Austin on a HiSeq 2000 (Illumina) on a 2×100 paired end run. All sequences are deposited with the National Center for Biotechnology Information Sequence Read Archive under Accession Number SRP033652.

### RNA-seq Illumina library preparation

To prepare RNA for RNA-seq sequencing libraries, cell pellets or ∼100 mg sections of wound tissue were homogenized 2–4 times in a Mini-Beadbeater in 1 mL RNA Bee (Tel-Test) in 2 mL vials with Lysing Matrix B, and aqueous phases of extractions from different sections of the same wound were pooled before continuing with the extraction. RNA was then prepared according to the RNA Bee manufacturer's protocol. DNA contamination was then removed by DNAse digestion as described [31]. rRNA integrity was then verified by agarose gel electrophoresis. Starting with 5 µg of total RNA, bacterial rRNA was depleted from all samples with the Ribo-Zero rRNA Removal Kit (Bacteria) (Epicentre), and then mammalian rRNA was depleted from all samples with the Ribo-Zero Gold Kit (Human/Mouse/Rat) (Epicentre) according to the manufacturer's protocol. Remaining RNA was fragmented with the NEBNext Magnesium RNA Fragmentation Module (New England Biolabs) according to the manufacturer's protocol with a 5′ incubation time. Illumina sequencing libraries were then prepared with the NEBNext Multiplex Small RNA Library Prep Set for Illumina (New England Biolabs) according to the manufacturer's protocol. Finished sequencing libraries were size selected on a polyacrylamide gel for fragments ∼140–300 bp. Libraries were sequenced at the Genome Sequencing and Analysis Facility at the University of Texas at Austin on a HiSeq 2000 (Illumina) on either a 1×100 single end or a 2×100 paired end run. All sequences are deposited with the National Center for Biotechnology Information Sequence Read Archive under Accession Number SRP033652.

### RNA-seq bioinformatic analyses

RNA-seq reads were analyzed and differential gene expression was determined with the R package DESeq [53] largely as described [31] with the following modifications: the *P. aeruginosa* PAO1 genome (GenBank accession no. AE004091.2) was used for read alignment, and COGs used for enrichment analyses were obtained from the *Pseudomonas* Genome Database [54]. P values given for differentially expressed genes are adjusted for multiple testing. Enrichment of differentially regulated genes in a given COG category was determined by comparing the prevalence of up- or down-regulated genes assigned to a specific COG category to the prevalence of genes in the entire genome assigned to that COG category using Fisher's exact test.

### Tn-seq bioinformatics analyses

Tn-seq reads were parsed, mapped, and tallied, and differential mutant abundance was determined using a custom Unix, Perl, and R pipeline (available at http://github.com/khturner/Tn-seq). First, reads containing the 12-bp transposon T8 end sequence 5′-TATAAGAGTCAG-3′ were identified (allowing for 1 mismatch or indel) using fqgrep (http://github.com/indraniel/fqgrep), and sequence up to and including the transposon end sequence were removed with the modified Perl script called “trimmer”. The remaining sequence was then mapped to the *P. aeruginosa* PAO1 genome (GenBank accession no. AE004091.2) using bowtie version 2.10 [55], and individual insertion sites and the number of reads originating from them were tallied with the Unix script “TnSeq.sh”. The data analysis method, contained in the Unix script “TnSeqAnalysis.sh” and the R script “TnSeqDESeq.R”, was inspired largely by the ESSENTIALS software package [56], and is described in detail below. After removing the 50 most abundant insertion sites from analysis to correct for amplification bias, insertion location vs. number of reads data was smoothed using locally weighted LOESS smoothing using a smoothing parameter (α) of 1 to correct for genomic position-dependent effects on apparent mutant abundance. Then, this data was normalized using DESeq [53] with default parameters. For gene knockout abundance analysis, a modified annotation was generated with the 3′ 10% of every gene removed (to ignore insertions that may not abolish gene function). Then, the smoothed, normalized number of transposon-derived reads per gene and the number of insertions mapping to each gene was tallied using this modified annotation in R. The number of transposon-derived reads was incremented by one for each gene to avoid dividing by zero when comparing to a condition with no mutants detected. Finally, differential mutant abundance was calculated using a negative binomial test with DESeq, artificially setting normalization factors to 1 (because the data was normalized per insertion).

## Supporting Information

Figure S1 *P. aeruginosa* global gene expression and knockout fitness in chronic wound infection. Log_2_-transformed fold change gene expression (y axis) and knockout abundance (x axis) of *P. aeruginosa* in murine chronic wound infections as compared to growth in MOPS-succinate (Succ.). Significant (Sig.) changes in gene expression (fold change ≥4, P<0.01, negative binomial test) and mutant abundance (fold change ≥4, P<0.05, negative binomial test) are colored as shown (N.C., no change).
(TIF)

Figure S2 Correlation between gene expression and knockout fitness is not improved for highly regulated or conditionally essential genes. (A, B, and C) Spearman rank correlation coefficient between fold change expression (F.C. Expr.) and fold change mutant abundance (F.C. Mut. Abund.) in either (A) the burn wound-MOPS-succinate comparison, (B) the chronic wound-MOPS-succinate comparison, or (C) the chronic wound-burn wound comparison (y axis) as a function of the degree of up-regulation (solid line) or fitness defect (dashed line) (x axis). Only genes with transposon-derived Tn-seq reads were considered.
(TIF)

Figure S3 *In vitro* characterization and complementation of individual mutants constructed for this study. (A) An *faoA* deletion mutant cannot catabolize long chain fatty acids. Shown are cultures of the indicated strains inoculated at 1∶100 dilution from a MOPS-succinate (MOPS-Succ) overnight culture in the indicated media after 6.5 hours of growth. Plasmid pFaoA carries *faoA* in a pUCP18 vector backbone (see Materials and Methods). (B) Deletion mutants tested in Figure 5B are auxotrophic. Shown are overnight cultures of the indicated strains grown in MOPS-succinate. Auxotrophies were verified by the addition of 100 µg/mL histidine (+His), 100 µg/mL each isoleucine, leucine, and valine (+Ile, Leu, Val), 10 µM p-aminobenzoate (+PABA), or 10 µg/mL each adenine, guanine, xanthine, and hypoxanthine (+purines). Plasmids pPabC and pPurF carry the *pabC* and *purF*, respectively, in a pUCP18 vector backbone (see Materials and Methods). (C) A *cheR1* mutant cannot chemotax. Shown is a semisolid (0.3% agar) LB plate supplemented with 150 µg/mL carbenicillin into which the designated strain has been stabbed. Plasmid pCheR1 carries *cheR1* in a pUCP18 vector backbone (see Materials and Methods). Examination by light microscopy indicated that PAO1 Δ*cheR1* does not exhibit a gross swimming defect (data not shown).
(TIF)

Table S1 RNA-seq sequencing and analysis information.
(DOCX)

Table S2 *P. aeruginosa* PAO1 relative gene expression in burn and chronic wounds as compared to MOPS-succinate.
(XLSX)

Table S3 *P. aeruginosa* PAO1 virulence gene expression in burn and chronic wounds as compared to MOPS-succinate.
(XLSX)

Table S4 Tn-seq sequencing and analysis information.
(DOCX)

Table S5 *P. aeruginosa* PAO1 relative mutant abundance in burn and chronic wounds as compared to MOPS-succinate.
(XLSX)

Table S6 *P. aeruginosa* PAO1 virulence gene mutant abundance in burn and chronic wounds as compared to MOPS-succinate.
(XLSX)

Table S7 Fitness contribution of biosynthetic genes in both burn and chronic wounds as compared to MOPS-Succinate.
(XLSX)

Table S8 *P. aeruginosa* PAO1 relative gene expression and relative mutant abundance in chronic wounds as compared to burn wounds.
(XLSX)
