# Correction: Requirements for *Pseudomonas aeruginosa* Acute Burn and Chronic Surgical Wound Infection

**DOI:** 10.1371/journal.pgen.1004743

**Published:** 2014-10-17

**Authors:** The PLOS Genetics Staff

The Funding statement for this article is incorrect. The correct Funding statement is provided below:

**Funding:** This work was supported by grant W911NF-13-1-0199 from the US Army Research Office. KHT is a Cystic Fibrosis Foundation Postdoctoral Research Fellow. MW is a Burroughs Wellcome Investigator in the Pathogenesis of Infectious Disease. The funders had no role in study design, data collection and analysis, decision to publish, or preparation of the manuscript.

## References

[1] Turner KH, Everett J, Trivedi U, Rumbaugh KP, Whiteley M (2014) Requirements for *Pseudomonas aeruginosa* Acute Burn and Chronic Surgical Wound Infection. PLoS Genet 10(7): e1004518 doi:10.1371/journal.pgen.1004518 25057820 10.1371/journal.pgen.1004518 PMC4109851

