# Supplementary figures and images for: Requirements for Pseudomonas aeruginosa Acute Burn and Chronic Surgical Wound Infection
Source: PLoS Genet. 2014 Jul 24;10(7):e1004518. doi: 10.1371/journal.pgen.1004518 (PMC4109851; doi:10.1371/journal.pgen.1004518)

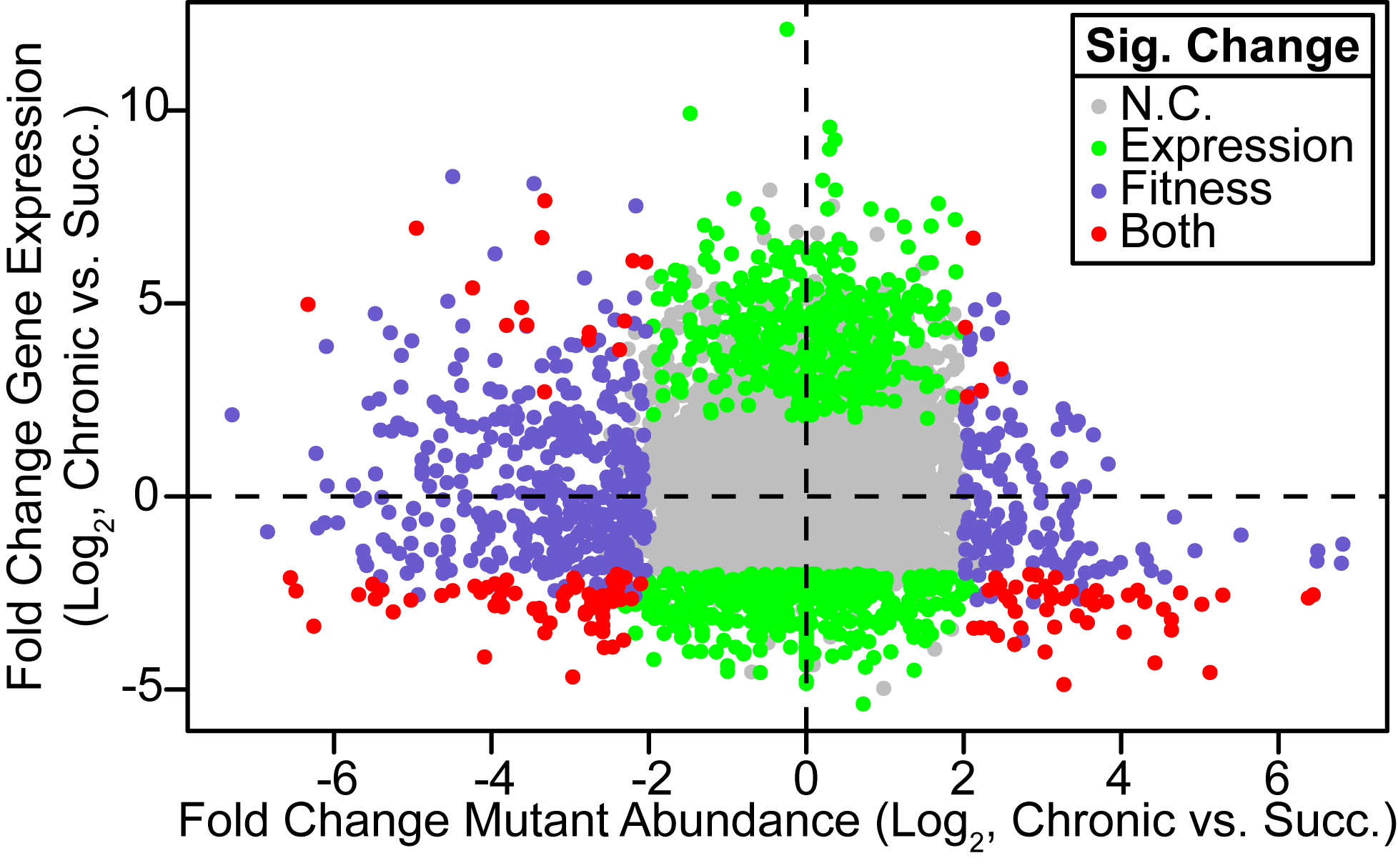

Supplement: Figure S1 — P. aeruginosa global gene expression and knockout fitness in chronic wound infection. Log2-transformed fold change gene expression (y axis) and knockout abundance (x axis) of P. aeruginosa in murine chronic wound infections as compared to growth in MOPS-succinate (Succ.). Significant (Sig.) changes in gene expression (fold change ≥4, P<0.01, negative binomial test) and mutant abundance (fold change ≥4, P<0.05, negative binomial test) are colored as shown (N.C., no change). (TIF) [file pgen.1004518.s001.tif]

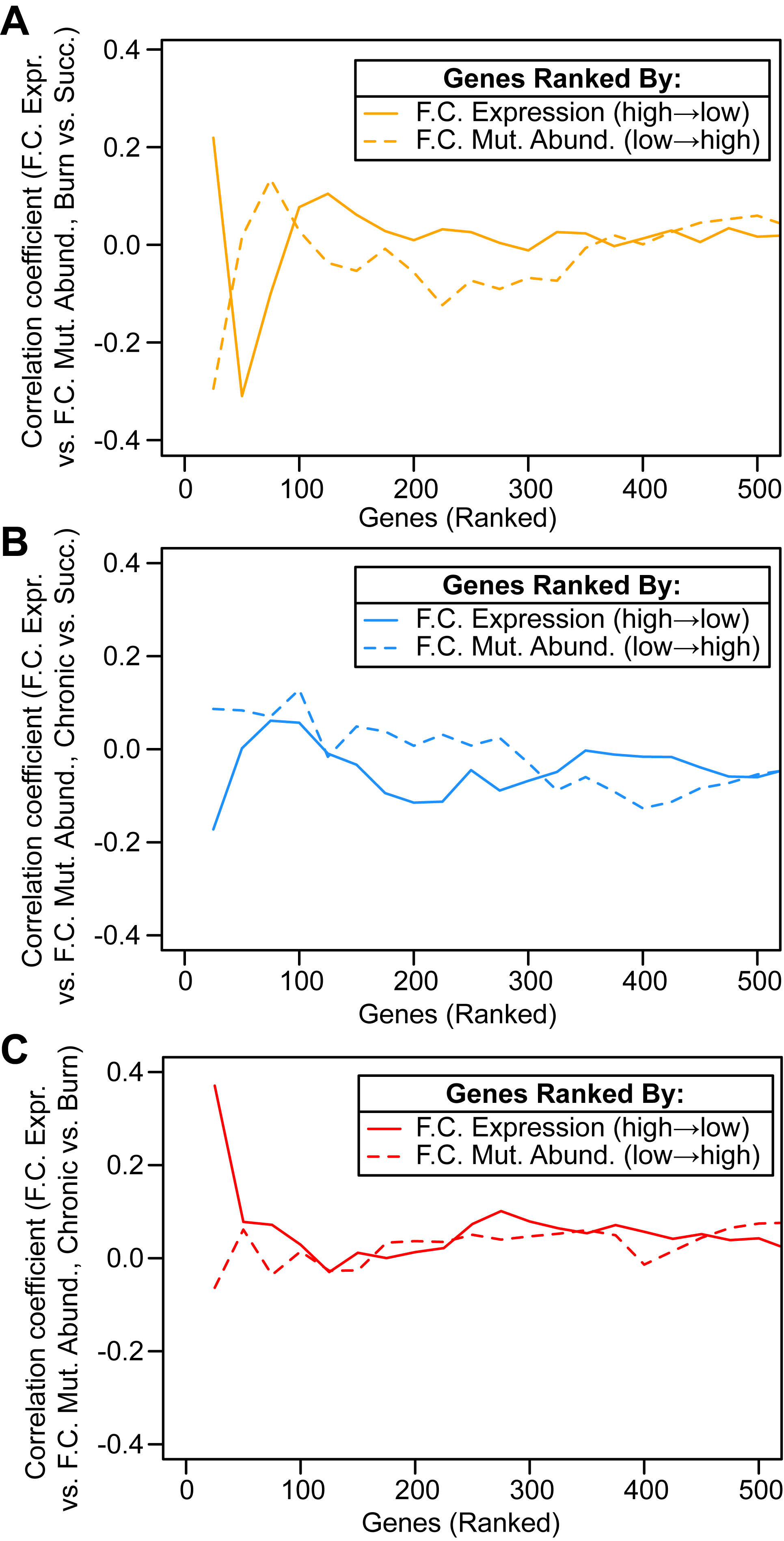

Supplement: Figure S2 — Correlation between gene expression and knockout fitness is not improved for highly regulated or conditionally essential genes. (A, B, and C) Spearman rank correlation coefficient between fold change expression (F.C. Expr.) and fold change mutant abundance (F.C. Mut. Abund.) in either (A) the burn wound-MOPS-succinate comparison, (B) the chronic wound-MOPS-succinate comparison, or (C) the chronic wound-burn wound comparison (y axis) as a function of the degree of up-regulation (solid line) or fitness defect (dashed line) (x axis). Only genes with transposon-derived Tn-seq reads were considered. (TIF) [file pgen.1004518.s002.tif]

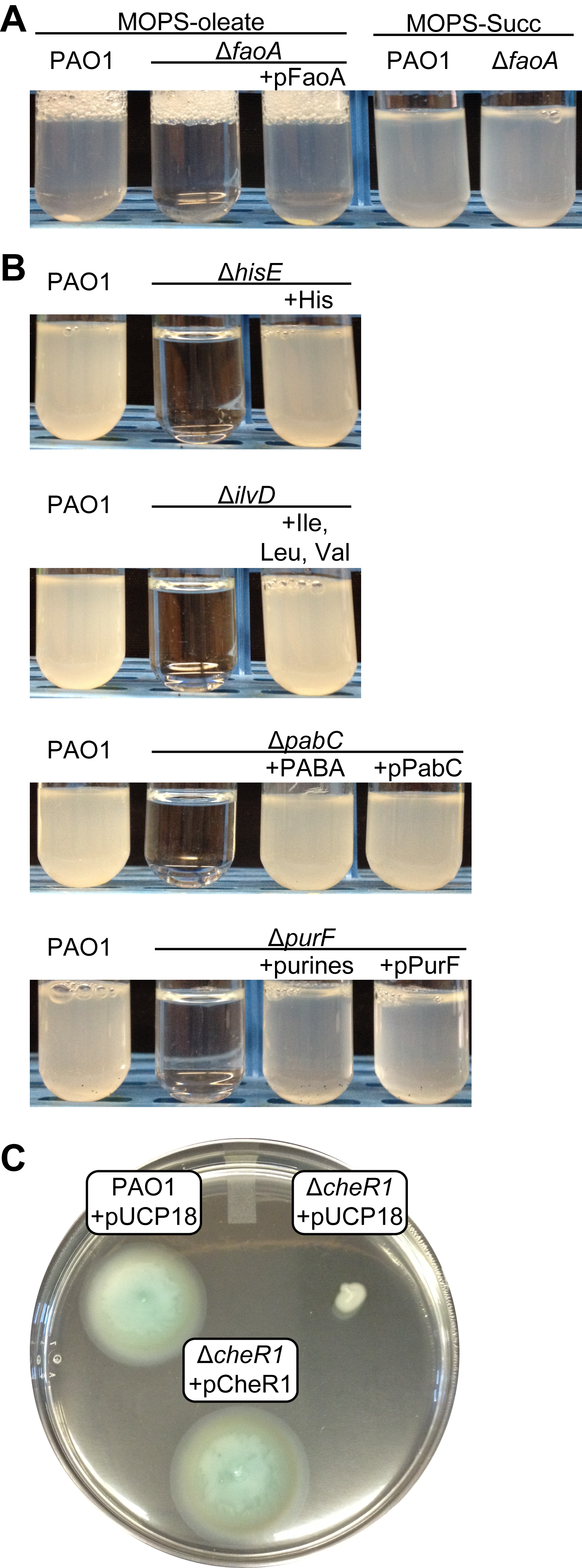

Supplement: Figure S3 — In vitro characterization and complementation of individual mutants constructed for this study. (A) An faoA deletion mutant cannot catabolize long chain fatty acids. Shown are cultures of the indicated strains inoculated at 1∶100 dilution from a MOPS-succinate (MOPS-Succ) overnight culture in the indicated media after 6.5 hours of growth. Plasmid pFaoA carries faoA in a pUCP18 vector backbone (see Materials and Methods). (B) Deletion mutants tested in Figure 5B are auxotrophic. Shown are overnight cultures of the indicated strains grown in MOPS-succinate. Auxotrophies were verified by the addition of 100 µg/mL histidine (+His), 100 µg/mL each isoleucine, leucine, and valine (+Ile, Leu, Val), 10 µM p-aminobenzoate (+PABA), or 10 µg/mL each adenine, guanine, xanthine, and hypoxanthine (+purines). Plasmids pPabC and pPurF carry the pabC and purF, respectively, in a pUCP18 vector backbone (see Materials and Methods). (C) A cheR1 mutant cannot chemotax. Shown is a semisolid (0.3% agar) LB plate supplemented with 150 µg/mL carbenicillin into which the designated strain has been stabbed. Plasmid pCheR1 carries cheR1 in a pUCP18 vector backbone (see Materials and Methods). Examination by light microscopy indicated that PAO1 ΔcheR1 does not exhibit a gross swimming defect (data not shown). (TIF) [file pgen.1004518.s003.tif]
