# Supplementary material for: Requirements for Pseudomonas aeruginosa Acute Burn and Chronic Surgical Wound Infection
Source: PLoS Genet. 2014 Jul 24;10(7):e1004518. doi: 10.1371/journal.pgen.1004518 (PMC4109851; doi:10.1371/journal.pgen.1004518)
Supplement: Table S1 — RNA-seq sequencing and analysis information. (DOCX) [file pgen.1004518.s004.docx]

**Table S1. RNA-seq sequencing and analysis information.**

| **Sample** | **Replicate** | **Total reads** | **Reads after adapter trimming (%*)** | **Reads mapping to PAO1 genome (%*)** | **Reads mapping to non-rRNA/tRNA genes (%*)** | **Average reads/gene** |
| --- | --- | --- | --- | --- | --- | --- |
| MOPS-Succinate | 1 | 32,718,002 | 29,594,835 (90.5%) | 25,724,696 (86.9%) | 15,680,708  (61.0%) | 2797 |
|  | 2 | 25,802,585 | 23,442,607 (90.9%) | 20,718,403 (88.4%) | 14,797,721  (71.4%) | 2640 |
| Burn Wound | 1 | 24,463,822 | 20,670,299 (84.5%) | 4,963,574 (24.0%) | 1,833,857  (36.9%) | 327 |
|  | 2 | 74,478,829 | 59,268,119 (79.6%) | 10,925,868 (18.4%) | 3,780,015  (34.6%) | 674 |
| Chronic Wound | 1 | 74,137,453 | 59,537,704 (80.3%) | 1,647,275  (2.8%) | 630,513  (38.3%) | 112 |
|  | 2 | 22,587,153 | 18,343,956 (81.2%) | 739,401  (4.0%) | 215,119  (29.1%) | 38 |

*Expressed as a percent relative to the previous column.
