# Supplementary material for: Requirements for Pseudomonas aeruginosa Acute Burn and Chronic Surgical Wound Infection
Source: PLoS Genet. 2014 Jul 24;10(7):e1004518. doi: 10.1371/journal.pgen.1004518 (PMC4109851; doi:10.1371/journal.pgen.1004518)
Supplement: Table S4 — Tn-seq sequencing and analysis information. (DOCX) [file pgen.1004518.s007.docx]

**Table S4. Tn-seq sequencing and analysis information.**

| **Sample** | **Replicate** | **Total reads** | **Reads with Tn end sequence (%*)** | **Reads mapping to PAO1 genome (%*)** | **Number of Tn insertion sites identified** | **Average reads/site** |
| --- | --- | --- | --- | --- | --- | --- |
| MOPS-Succinate | 1 | 40,631,383 | 5,212,457 (12.8%) | 4,524,052 (86.8%) | 100,971 | 45 |
|  | 2 | 50,131,688 | 5,953,730 (11.9%) | 4,260,960 (71.6%) | 104,015 | 41 |
| Burn Wound | 1 | 94,547,593 | 2,592,455 (2.7%) | 1,669,957 (64.4%) | 26,768 | 62 |
|  | 2 | 44,409,650 | 4,337,799 (9.8%) | 3,419,265 (78.8%) | 62,517 | 55 |
| Chronic Wound | 1 | 269,135,273 | 757,534  (0.3%) | 454,353  (60.0%) | 34,428 | 13 |
|  | 2 | 116,676,167 | 730,961  (0.6%) | 521,899  (71.4) | 42,163 | 12 |

*Expressed as a percent relative to the previous column.
